# A Case of Duodenal Resection and Duodenojejunostomy for Multiple Small Bowel Infarction in Patient With Inherited Thrombophilia and Vitamin K Antagonist Induced Critical Hypocoagulation

**DOI:** 10.7759/cureus.13129

**Published:** 2021-02-04

**Authors:** Badri Kobalava, Anzor Kvashilava, Giorgi Giorgobiani, Irina G Datikashvili-David, Nana Turava

**Affiliations:** 1 Medical School, Course of Surgery, New Vision University, Tbilisi, GEO; 2 Surgery Department #3, Faculty of Medicine, Tbilisi State Medical University, Tbilisi, GEO; 3 Surgery Division, Aversi Clinic, Tbilisi, GEO; 4 Hematology, Aversi Clinic, Tbilisi, GEO; 5 Radiology, Aversi Clinic, Tbilisi, GEO

**Keywords:** small intestine, duodenojejunostomy, intestinal infarction, clotting disorders, factor v leiden, thrombophilia, venous thrombosis, apc resistance, anticoagulation, duodenal resection

## Abstract

We present a case of the multiple venous intestinal infarction in patient with two inherited thrombophilias: Leiden factor V (LFV) and factor VIII elevation. The patient had a critical hypocoagulation caused by vitamin K antagonist (VKA) overdose. At laparotomy, several intestinal segments were necrotic and ischemic. Coagulopathy was corrected by the transfusion of the fresh frozen plasma. Because of the 4th duodenal segment infarction distal segmental duodenectomy with side-to-side duodenojejunostomy was done, which is a rarely performed procedure. On postoperative day 6 deep vein thrombosis developed, despite nadroparin profillaxes, early mobilisation and compressive stockings.

Our case demonstrated that in patients with congenital thrombophilia, development of the mesenteric venous thrombosis is possible even with VKA induced severe hypocoagulation. Venous infarction of the small bowel can be associated with the hemoperitoneum and gastrointestinal bleeding. After resection of the fourth duodenal segment, side-to-side duodenojejunostomy is a feasible method of reconstruction.

## Introduction

The overall incidence of venous thrombosis is about 1.43 per 1,000 person a year [1]. Leiden factor V (LFV) thrombophilia is the most common congenital cause of venous thromboembolism [2]. The mutation affects protein C structure, which normally inactivates coagulation factors V and VIII thus exhibiting anticoagulant properties. The mutation results in the decreased anticoagulation effect of the activated protein C and thrombophilia. Numerous authors report the role of the LFV in the development of deep vein thrombosis, pulmonary embolism, ischemic stroke, myocardial infarction, splenic infarction, etc. Publications regarding intestinal infarction are very rare [3]. Factor VIII is plasma sialoglycoprotein which has a key role in hemostasis. Its elevation. Its deficiency leads to hemophilia, while elevation (> 150% of normal) is a risk for venous thrombosis [4].

## Case presentation

The 73-year-old man, white, of European descent, was hospitalized for acute recurrent abdominal pain, nausea, multiple vomiting with a large amount of coffee-ground masses, inability to pass flatus and feces, and fatigue. It was a fifth day from the onset of symptoms.

He had a history of spontaneous deep vein thrombosis with pulmonary artery embolism. LFV (G1691A heterozygous mutation) and factor VIII elevation (225%) was diagnosed and warfarin was prescribed to keep INR between 2 and 3. He used to check the coagulation state regularly once a month.

The patient had several episodes of abdominal pain and vomiting before. Those attacks had been resolved spontaneously. He has been taking tamsulosin for benign prostatic hyperplasia and atorvastatin for correction of dyslipidemia. In history, 17 and 15 years ago he was operated on for posttraumatic pelvic bone and femur fracture. 

**Figure 1:**
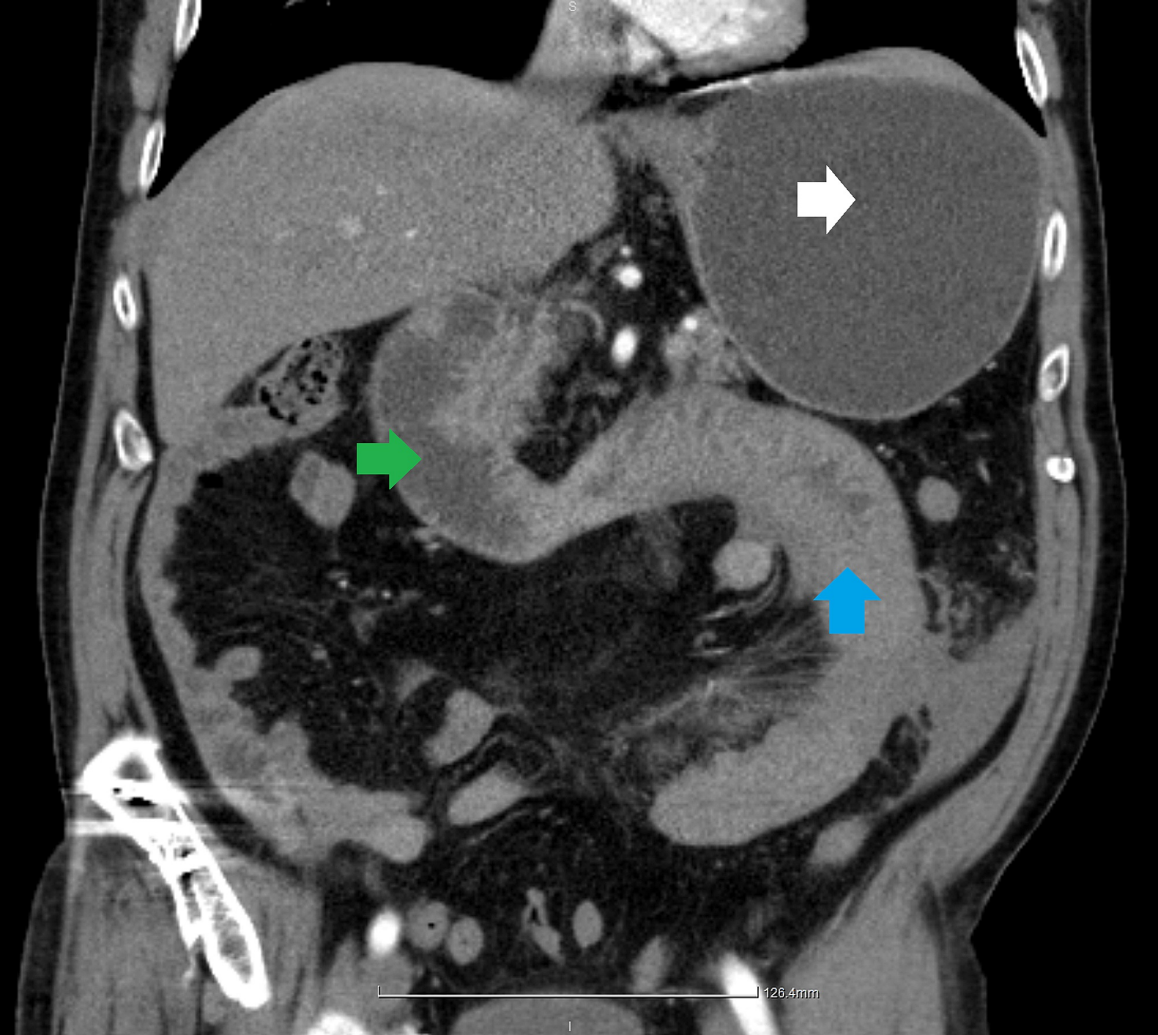
CT sagittal reconstruction. The stomach (white arrow) and duodenum (green arrow) are distended. The distal duodenum, DJ junction, and initial jejunum (blue arrow) are swollen.

At admission, the patient was pale, weak, covered with sweat, hypotensive and tachycardic. The abdomen was distended and sharply painful. Peritoneal signs were positive. The amount of the coffee-ground vomit was significant. Blood tests at admission: WBC 17.6 10/l; CRP 601 nmol/l; aPTT 162 sec; INR was beyond the range of the analyzer. After the administration of the fresh frozen plasma, preoperative INR was 4.27. CT scan revealed distended stomach and duodenum, several local swellings of the intestinal wall, and free fluid in the abdominal cavity (Figure 1).

**Figure 2:**
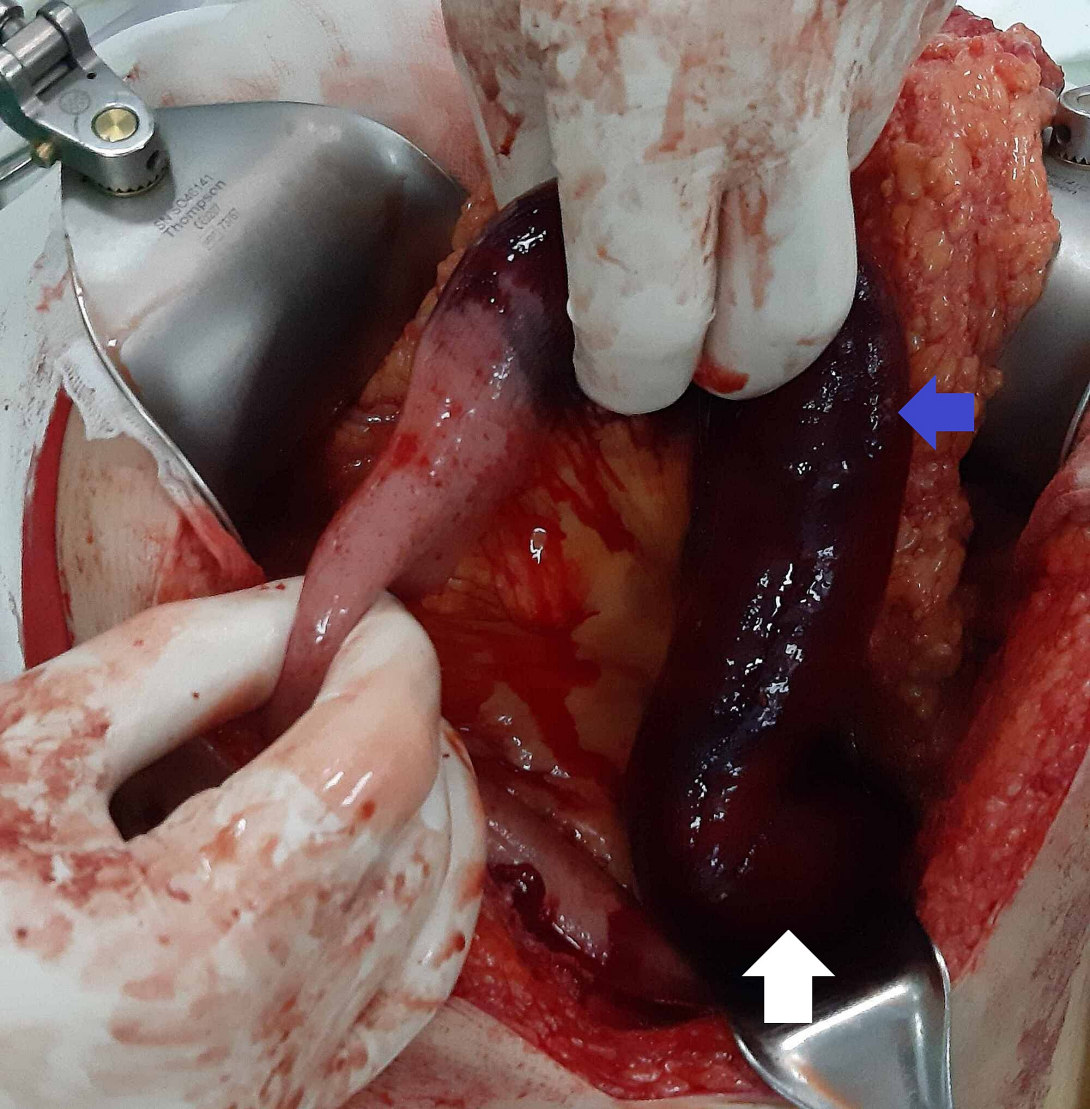
Laparotomy revealed hemoperitoneum. Fourth portion of the duodenum (white arrow) and initial jejunum (blue arrow) are necrotic.

**Figure 3:**
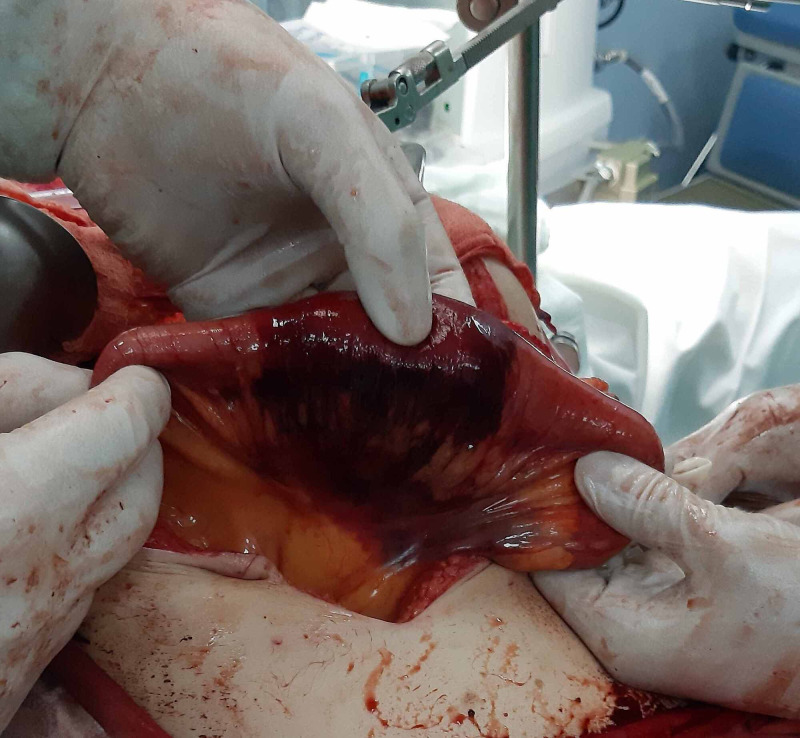
Second necrotic jejunal segment.

**Figure 4:**
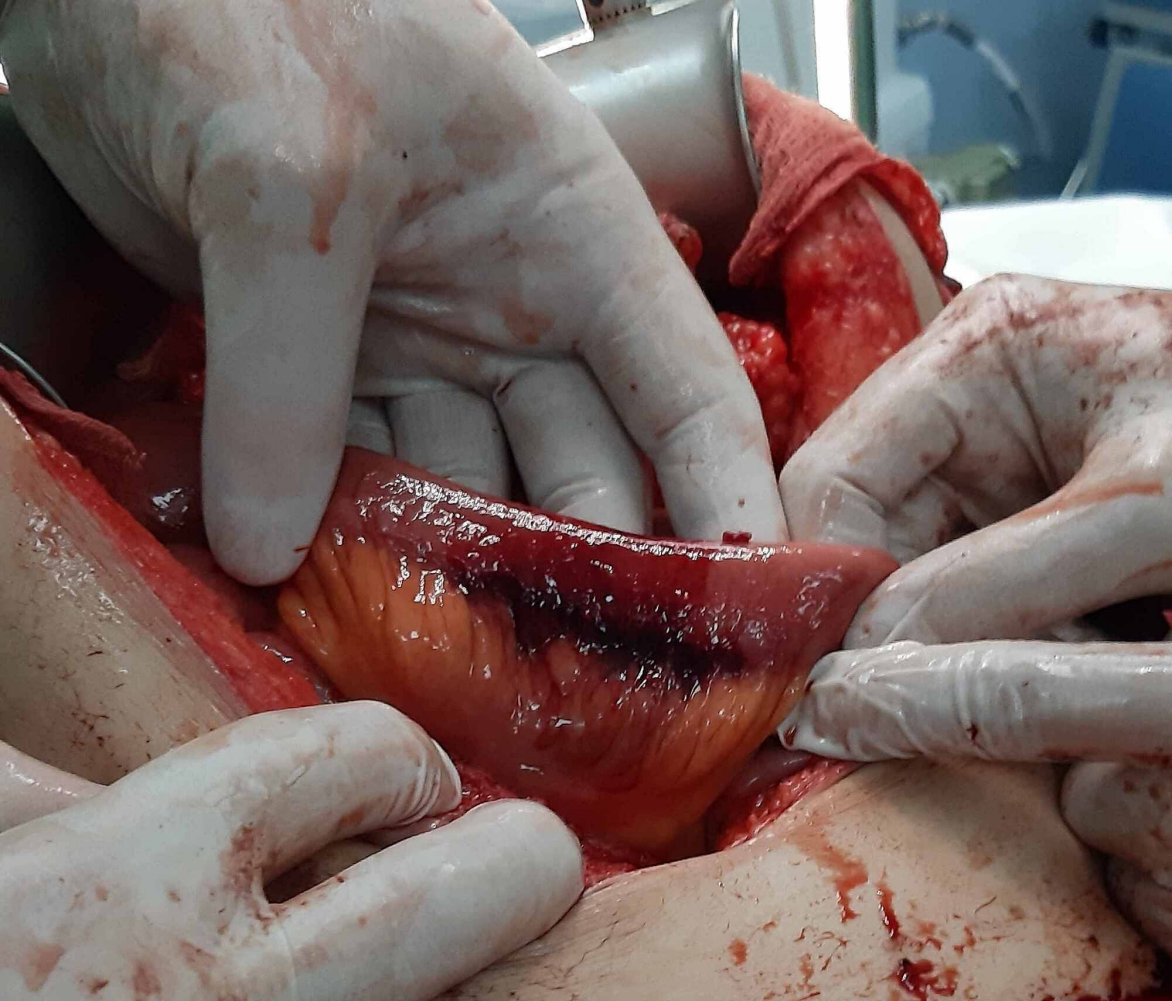
Ischemic segment of the ileum.

At laparotomy, the hemoperitoneum was found. The fourth part of the duodenum and the initial 40cm of the jejunum was necrotic (Figure 2). Another 7 cm necrotic segment was found 30 cm distally (Figure 3). The third 6 cm long segment was ischemic with bruises in the intestinal wall and mesentery but still viable (Figure 4).

After the takedown of the hepatic flexure of the colon, the duodenum was mobilized by the Kocher maneuver. The healthy 3rd portion of the duodenum was isolated, dissecting it underneath the mesenteric root from the right. After, the fourth duodenal segment was dissected from the left side in the same manner, transecting the Treitz ligament. Necrotic initial jejunum was completely mobilized and retrieved to the left side of the mesenteric root. The side-to-side duodenojejunal anastomosis was formed on the anterior surface of the second part of the duodenum. After, the third part of the duodenum was transected at the viable area using a GIA stapler. 

**Figure 5:**
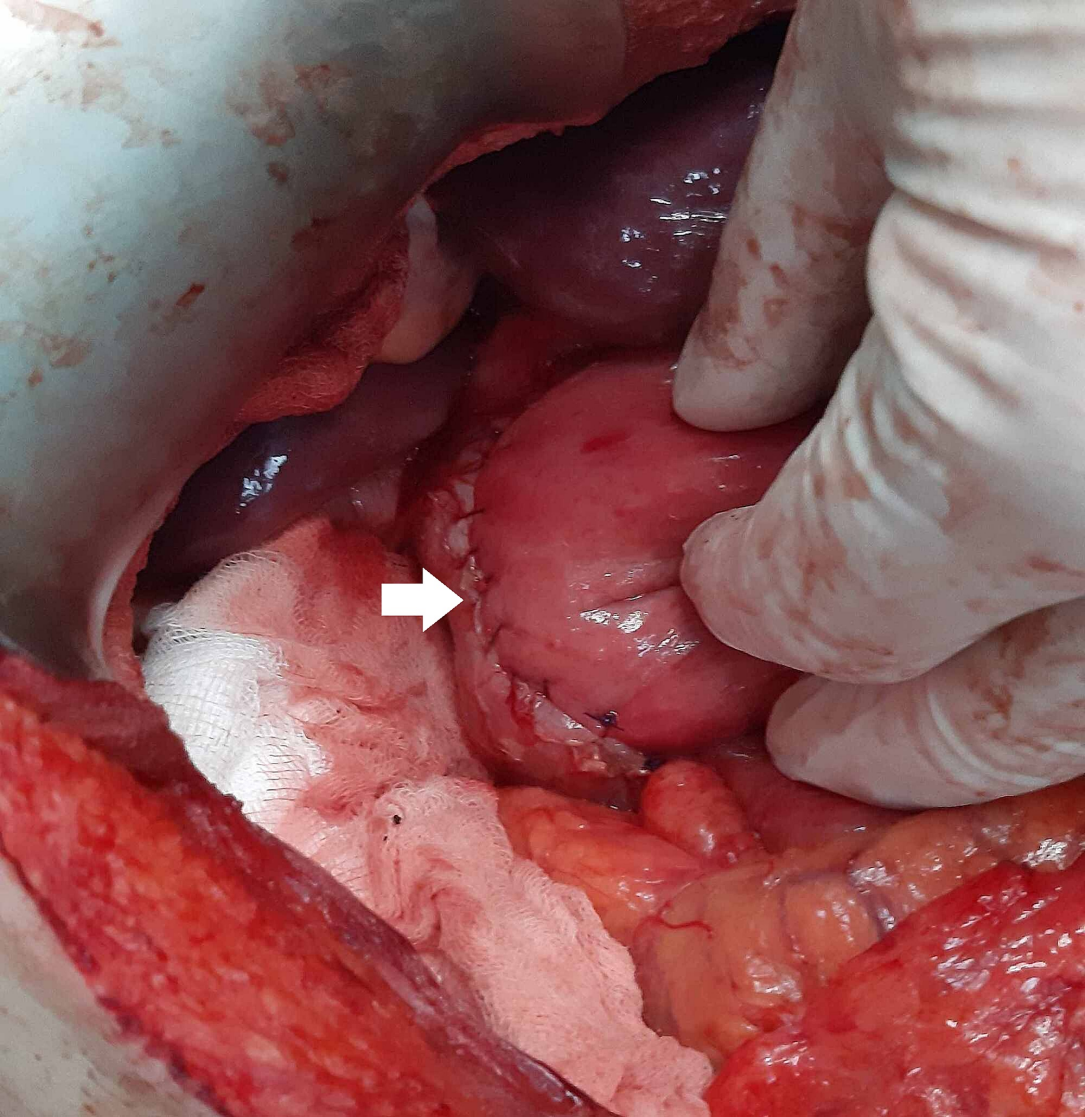
Side-to-side duodenojejunostomy (arrow).

Another necrotic jejunal segment was resected and side-to-side jejunojejunostomy was created (Figure 5). The third ischemic segment of the intestine was left unresected.

After surgery, the patient was placed in the ICU. Anticoagulation was postponed due to a critical hypocoagulation state. Coagulopathy was corrected by fresh frozen plasma transfusion. Tracheal extubation was done on the first postoperative day (POD). Oral fluids allowed on POD2. On the third POD3 the patient was transferred to wards, 7600 IU of nadroparin (fraxiparine) was started, and progressive ambulation began. Liquid and semi-liquid meal were allowed on POD5, after the restoration of the bowel peristalsis. On the morning of the POD6 the patient complained of leg pain. Doppler ultrasound revealed deep vein thrombosis, despite Nadroparin prophylaxis, early mobilization, and compressive stockings. Its dose was increased up to 11,400 IU and walking was restricted. On POD13 the patient was discharged from the hospital after a doppler ultrasound recheck. 11,400 IU of nadroparin was prescribed for three weeks. After, it was replaced with 20 mg of rivaroxaban daily. After one and three months from surgery, the patient was uneventful.

## Discussion

About 15% of all intestinal infarction cases are caused by thrombosis of the mesenteric vein [5]. 20%-30% of them are induced by inherited diseases like LFV, protein S deficiency, protein C deficiency, prothrombin mutation, antiphospholipid syndrome, and antithrombin deficiency [6]. About 20% of cases are idiopathic. Other reasons are trauma, portal hypertension, inflammatory bowel disease, acute pancreatitis, sepsis [7]. Factor VIII elevation further increases the risk of venous thrombosis. It is higher than for arterial thrombosis and the risk depends on the level of the factor VIII [8-10].

Anticoagulant therapy for the prevention of recurrent thrombosis is very effective [11] but cases of failure of such prophylaxis are well known [11,12]. Risk of the recurrent VTE thrombosis during the long-term VKA treatment is 18 per 1000 according to Cochrane meta-analysis [13].

Thrombosis despite anticoagulation could be caused by some diseases: pemphigus vulgaris [14], cancer [15], antiphospholipid antibody syndrome, obesity, a neurologic disease with extremity paresis, pregnancy, drug administration (e.g., oral contraceptives), etc. or inherited disorders: antithrombin deficiency, protein C deficiency, protein S deficiency, LFV. The last one has the highest prevalence (3%-7%), Incidence (12%-20%), and recurrence rate (50%) in whites of European ancestry [16].

Traditionally vitamin K antagonists (VKAs) are used for the prevention of recurrent thrombosis in high-risk patients, but modern data suggest high efficacy of the direct oral anticoagulants in inherited thrombophilia [16,17]. Besides, they have several advantages: no need for the regular laboratory monitoring of the INR, predictable dose-response, fewer drug and food interactions [18]. The patient was switched to rivaroxaban instead of warfarin.

Another unusual issue of our case was the necessity of the partial duodenectomy. Such intervention is rarely used. The main indication of the duodenal 3rd or 4th segment resection is a benign or malignant tumor of this area: generally adenoma, early-stage adenocarcinoma, or gastrointestinal stromal tumor [19]. Other indications are duodenal injury and peptic ulcer complicated with perforation or hemorrhage. Anatomic position of these D3/D4 behind the root of the small gut, relations to the pancreas and lower pancreaticoduodenal, and superior mesenteric vessels makes resection challenging. After resection, end-to-end, side-to-side, or end-to-side duodenojejunostomy might be established [20].

## Conclusions

The risk of venous thrombosis despite effective anticoagulation is significant in patients with LFV. Factor VIII elevation further increases the probability. Our case demonstrated that in the case of congenital thrombophilia, the development of the mesenteric venous thrombosis is possible even with VKA induced severe hypocoagulation. Venous infarction of the small bowel can be associated with hemoperitoneum and GI bleeding. After resection of the fourth duodenal segment, side-to-side duodenojejunostomy is a feasible method of reconstruction.
